# The dichotomous outcomes of TNFα signaling in CD4^+^ T cells

**DOI:** 10.3389/fimmu.2022.1042622

**Published:** 2022-11-16

**Authors:** Nikolaos Skartsis, Leonardo M. R. Ferreira, Qizhi Tang

**Affiliations:** ^1^ Division of Nephrology and Hypertension, Department of Medicine, Mayo Clinic, Rochester, MN, United States; ^2^ Mayo Clinic William J. von Liebig Center for Transplantation and Clinical Regeneration, Mayo Clinic, Rochester, MN, United States; ^3^ Department of Microbiology and Immunology, Medical University of South Carolina, Charleston, SC, United States; ^4^ Department of Regenerative Medicine and Cell Biology, Medical University of South Carolina, Charleston, SC, United States; ^5^ Hollings Cancer Center, Medical University of South Carolina, Charleston, SC, United States; ^6^ Department of Surgery, University of California, San Francisco, San Francisco, CA, United States; ^7^ Diabetes Center, University of California, San Francisco, San Francisco, CA, United States; ^8^ Gladstone University of California San Francisco (UCSF) Institute of Genome Immunology, University of California, San Francisco, San Francisco, CA, United States

**Keywords:** tumor necrosis factor alpha, tumor necrosis factor receptor, T effector cells, T regulatory cells, pleiotropism, activation, costimulation, autoimmunity

## Abstract

TNFa blocking agents were the first-in-class biologic drugs used for the treatment of autoimmune disease. Paradoxically, however, exacerbation of autoimmunity was observed in some patients. TNFa is a pleiotropic cytokine that has both proinflammatory and regulatory effects on CD4^+^ T cells and can influence the adaptive immune response against autoantigens. Here, we critically appraise the literature and discuss the intricacies of TNFa signaling that may explain the controversial findings of previous studies. The pleiotropism of TNFa is based in part on the existence of two biologically active forms of TNFa, soluble and membrane-bound, with different affinities for two distinct TNF receptors, TNFR1 and TNFR2, leading to activation of diverse downstream molecular pathways involved in cell fate decisions and immune function. Distinct membrane expression patterns of TNF receptors by CD4^+^ T cell subsets and their preferential binding of distinct forms of TNFα produced by a diverse pool of cellular sources during different stages of an immune response are important determinants of the differential outcomes of TNFa-TNF receptor signaling. Targeted manipulation of TNFa-TNF receptor signaling on select CD4^+^ T cell subsets may offer specific therapeutic interventions to dampen inflammation while fortifying immune regulation for the treatment of autoimmune diseases.

## Introduction

In the late 1970s, tumor necrosis factor alpha (TNFa) was discovered due to its tumoricidal activity, as bacterially contaminated neoplastic tumors would regress in size (1, 2). Over the ensuing years, TNFa was shown to have an important proinflammatory role in a variety of preclinical experimental models (3) and human disease settings (4). Several studies have established an association between genetic and protein processing defects in the NF-κB signaling pathway downstream of TNFa in human autoimmune and inflammatory diseases, such as systemic lupus erythematosus (SLE) (5), Sjögren’s syndrome (6), Crohn’s disease (7), ulcerative colitis (8) and rheumatoid arthritis (RA) (9). In the clinical arena, inhibition of TNFa signaling in otherwise therapy-resistant patients has led to improved treatment outcomes in a variety of immune-mediated diseases, such as RA (10), inflammatory bowel disease (11), adult-onset Still’s disease (12), and psoriasis (13). Surprisingly, however, some patients experienced new-onset autoimmune diseases, such as multiple sclerosis (MS), psoriasis, or lupus-like syndromes following administration of TNFa blocking agents (14–16). This led to an insurgence of studies shedding light on the immunomodulatory role that TNFa exerts on T cells (17, 18). It is now clear that TNFa is a multifaceted cytokine that has both proinflammatory and immunoregulatory roles. The differential outcomes of TNFa signaling depend on the (a) type of receptor TNFa binds to, (b) cell type carrying the specific TNF receptor, (c) cellular source of TNFa production, (d) phase of the immune response, and (e) type of regulatory T cell (Treg) responsible for suppression in each disease setting (**Table 1**). Given the latest advances in our understanding of the intricacies of TNFa signaling and its central role in auto- and allo-immunity, we are reviewing and critically appraising the literature and discussing potential opportunities to further develop precision medicine approaches for autoimmune diseases and transplant rejection.

**Table 1:** Factors that influence the impact of TNFa on the outcome of the immune response.

	Proinflammatory	Regulatory
**TNFR type**	TNFR1	TNFR2
**TNFR-expressing cell**	CD4^+^ Teff	Treg
**Cellular source of TNFa**	Myeloid	CD4+ Teff
**Timing of TNFa exposure**	Early	Late
**Predominant type of Treg cell subset responsible for suppression**	Peripheral Treg	Thymic Treg

## TNFa and TNFR overview

### Ligand and receptor interactions

TNFa is a cytokine mainly produced by immune cells, such as macrophages, CD4^+^ T cells, NK cells, neutrophils, mast cells, and eosinophils. Yet, it is also secreted by non-immune cells, including endothelial cells, fibroblasts, and neurons (19, 20). TNFa is generated in a precursor form as a 233 amino acid type II single-pass transmembrane protein anchored in the cell membrane that is assembled in homotrimers and acts in a paracrine manner (21–23). Known as transmembrane TNFa (tmTNFa, 26kDa), this precursor protein can act both as a ligand and as a receptor during cell-to-cell interactions, thereby mediating both forward and reverse signaling (24, 25). It therefore plays a pivotal role in local inflammation. tmTNFa is processed by TNFa converting enzyme (TACE, also known as ADAM17) to generate soluble TNFa (sTNFa, 17 kDa) homotrimers (26, 27). Following TACE-mediated cleavage, the cytoplasmic domain of tmTNFa is further processed by the aspartyl protease SPPL2b, which then translocates to the nucleus and mediates proinflammatory cytokine production (28–30). The released fragment of sTNFa mediates additional endocrine function of TNFa in remote sites *via* hematogenous circulation. The individual roles of sTNFa and tmTNFa in mediating the pathogenesis of autoimmunity has been studied in transgenic mice expressing a TACE resistant form of tmTNFa (31). Interestingly, these mice were protected from autoimmune phenomena at a higher rate than TNFa KO animals, highlighting both the pathogenetic role of sTNFa and the partially protective role of tmTNFa (31). TACE inhibition has since emerged as a targeted strategy to preserve favorable inhibitory tmTNFa downstream signaling while efficiently blocking sTNFa deleterious activity (32).

**Figure 1:**
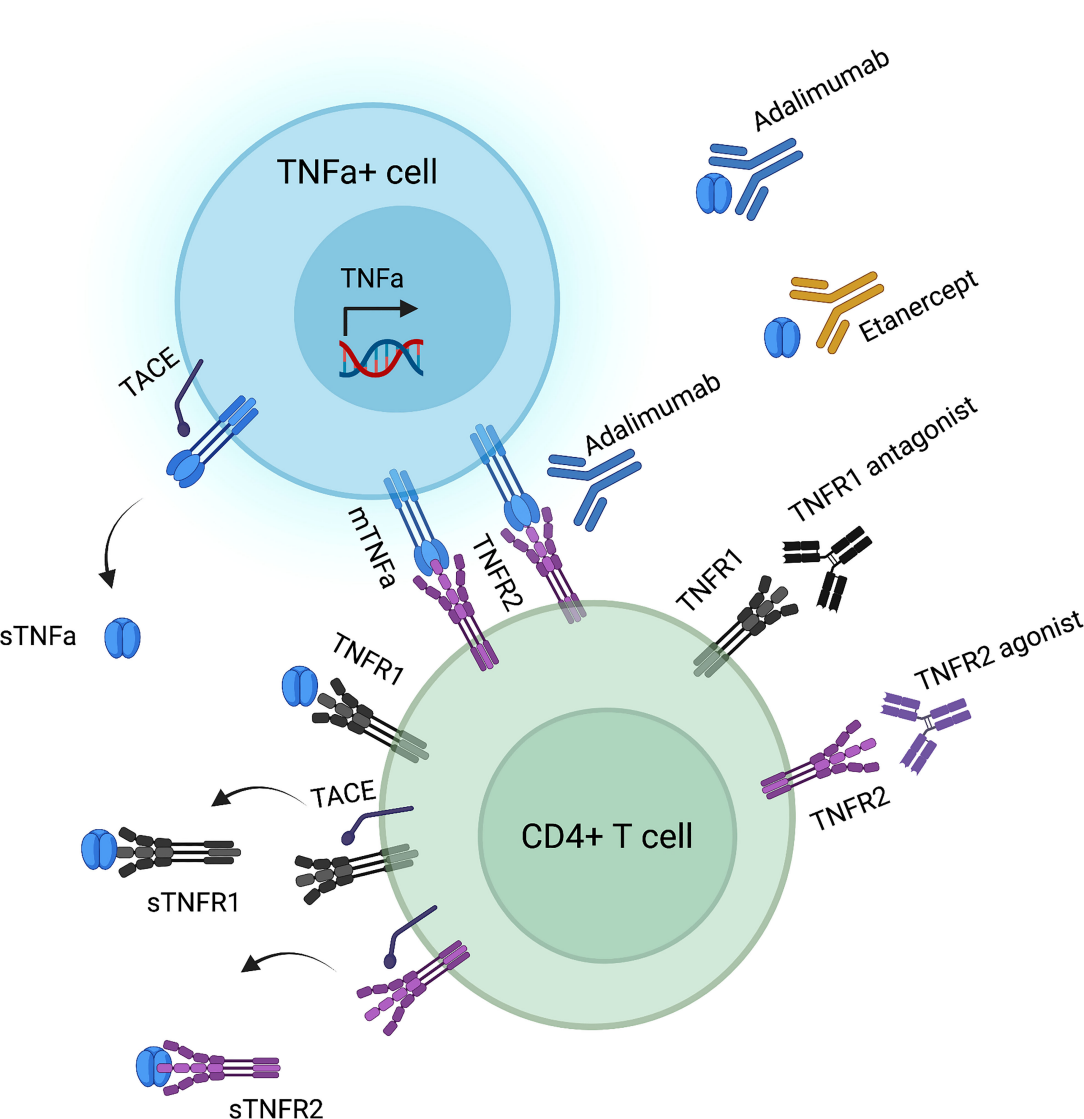
TNFa-TNFR interactions and corresponding biologic agents’ targets. Created by using biorender.com.

TNFa exerts its effects *via* binding to either TNFR1 or TNFR2 (**Figure 1**). TNFR1 is expressed in all nucleated cells in the form of pre-assembled trimers, while TNFR2 is preferentially expressed in immune cells, such as activated CD4^+^ effector T (Teff) and CD4^+^ Treg cells (33–35). Importantly, the assembly of TNFR trimers is ligand-independent and is regulated by cysteine-rich domains in the extracellular region of TNFR (36). Mutations in these domains that mediate receptor folding and trafficking lead to systemic inflammation called TNFR-associated periodic syndrome (TRAPS) (37–41). TRAPS-associated mutant TNFR1 is not secreted, therefore does not bind TNF, but, instead, is retained in the endoplasmic reticulum leading to less efficient induction of apoptosis, compared to wild-type TNFR1 (39). Upon binding of a TNFa homotrimer to a TNFR1 or a TNFR2 homotrimer, signaling is initiated after secondary assembly of initial TNFa-TNFR homotrimers (42, 43). Both TNF receptors can bind to both tmTNFa and sTNFa. Yet, tmTNFa has higher affinity to TNFR2 (44), while sTNFa has a more stable association with TNFR1 (45) (**Figure 1**).

### Signaling and pathway crosstalk

TNFR1 engagement triggers the transcription of proinflammatory genes *via* activation of the canonical NF-κB pathway (33, 46, 47). TNFR1 activation induces the recruitment of death domain (DD)-containing adapters, such as TNFR1-associated DD protein (TRADD) and receptor-interacting serine/threonine kinase 1 (RIPK1), with further recruitment of TNFR-associated factor 2 (TRAF2), culminating in the formation of signaling complex I. Associated cellular inhibitor of apoptosis (cIAP) 1/2 proteins ubiquitinate themselves and RIPK1, leading to further recruitment of TAK1/TAB2/TAB3 and linear ubiquitination assembly complex (LUBAC), which in concert polyubiquitinate NEMO and activate the IKK complex. This results in the phosphorylation and proteasomal degradation of IκB with the subsequent nuclear translocation of the active NF-κB complex (p50/p65). Additionally, TAK1 can lead to proinflammatory gene transcription *via* the phosphorylation of MAP kinases, such as the JNK and p38, which activate the AP-1 complex (48–50). Alternatively, TNFR1 signaling may lead to either apoptosis or necroptosis (51). Destabilization of complex I can lead to formation of complex II as non-ubiquinated RIPK1 and TRADD recruit FADD, C-FLIP and pro-caspase 8, ultimately leading to the activation of effector caspases (52). However, when caspase 8 is inhibited by caspase inhibitors or virally expressed proteins, RIPK1 and RIPK3 associate and either self-phosphorylate or phosphorylate each other, eventually leading to necroptosis (53).

TNFR2, in contrast to TNFR1, does not contain a death domain. Instead, TNFR2 directly recruits TRAF1 or TRAF2 along with cIAP1/2, triggering the recruitment of LUBAC, TAK1/TAB2/TAB3, and NEMO/IKK1/2 complexes, ultimately leading to activation of the canonical NF-kB pathway. tmTNFa binding to TNFR2 results in activation of the non-canonical NF-κB pathway *via* NIK accumulation, phosphorylation of IKK1 complex and processing of p100, and subsequent formation of p52/RelB heterodimers that translocate to the nucleus to activate transcription of target genes (54, 55). TNFR2 ligation in human Tregs enhances IL-2-induced proliferation mainly *via* the activation of the non-canonical NF-κB pathway (56). In addition to NF-κB pathway stimulation, TNFR2 crosslinking can also lead to MAPK activation. Indeed, p38 MAPK signaling is key to TNFR2-driven Treg activation and proliferation (57). On the other hand, pro-apoptotic signaling mediated by JNK activation depends on TNFR2 localization, which is regulated by TRAF2 (58) and requires the association of internalized TNFR2 with AIP1 (59).

TNFa binding to TNFR1 or TNFR2 may elicit downstream signaling that is not solely restricted to either type of receptor. Cross-talk between TNFR1 and TNFR2 may occur at multiple levels (60). Ligand passing has been credited with endowing TNFR2 the ability to deliver pro-apoptotic signals (61). According to this mechanism, owing to TNFR2’s more rapid association with TNFa and associated longer half-life of TNFa binding, TNFR2 increases the local TNFa concentration in the vicinity of TNFR1 receptors. These subsequently accept TNFa ligand molecules, inducing apoptosis (61). Under long-term TNFa exposure, TNFR1 and TNFR2 co-expressing cells prevent apoptosis by generating TRAF1 and TRAF2 heterodimers that are more efficient activators of NF-κB pathway signaling (62). Moreover, TNFR2 activation during inflammatory conditions may control TNFR1-induced activation *via* ASK1 ubiquitination (63).

## Role of TNFa-TNFR in T cell development in the thymus

TNFa has been found to be important in many stages of thymic T cell development as it promotes the apoptosis of triple negative CD3^-^CD4^-^CD8^-^ (64) and double positive CD4^+^CD8^+^ thymocytes (65). Beyond NF-κB’s conventional role in transmitting downstream TCR signals, TNFa can also directly upregulate anti-apoptotic genes, such as cIAP1/2, which is important for the maturation of CD4^+^ and CD8^+^ single-positive T cells during the later stages of T cell development (66). Contrary to their conventional counterparts, Treg development relies upon the cooperative activity of several TNF receptor superfamily members (67). Tregs are dedicated to suppressing immune responses, ensuring self-tolerance and immune homeostasis, limiting tissue damage by overactive immune responses (68). Their unique transcriptional program is bestowed on them by the sustained expression of the transcription factor FOXP3 (69). Thymic Treg (tTreg) development is a two-step process (70). First, TCR-CD28 signaling upregulates CD25 (IL2Rα) and CD122 (IL2Rβ) expression along with c-REL-dependent chromatin remodeling at the FOXP3 locus. Second, IL-2 signaling drives FOXP3 expression in a STAT5-dependent manner to endorse full Treg phenotype (70). TNFR2 expression is upregulated in Treg progenitors and serves as a link between TCR signaling strength and augmented IL-2/STAT5 signaling, eventually driving Treg differentiation (67).

## Effect on Teffs in the periphery

Teffs not only produce TNFa, but also respond to it. Cell-type specific ablation of TNFa expression showed that myeloid-derived TNFa mediates the pathogenesis of collagen-induced arthritis (CIA), whereas T cell-derived TNFa is protective during the induction phase of arthritis by limiting T cell priming and memory T cell development (71). Interestingly, in a preclinical model of EAE, TNF produced by myeloid cells exacerbated neuroinflammation by driving the recruitment of inflammatory cells in the central immune system (CNS), and TNF produced by T cells further promoted myeloid cell recruitment into the CNS (72). However, in secondary lymphoid organs, TNF derived from myeloid and T cells synergized to dampen encephalitogenic Th1 and Th17 responses by decreasing IL-12 and IL-6 production (72). Another study using CD4^+^CD45RB^hi^ T cell-induced colitis in lymphopenic mice showed that resident non-T cells are induced by Teffs *in situ* to produce TNFa, which in turn induced colitis. Of note, TNFa derived from Teffs was neither necessary nor sufficient to induce colitis (73). The exact nature of cell-to-cell interactions or soluble factors that Teffs employ to induce TNFa production by intestinal resident cells remains unclear. To address this, a subsequent study showed that TNFR2-deficient Teffs failed to induce full-fledged colitis in Rag1 KO mice due to their impaired capacity to produce Th1 cytokines, owing to increased p100/p52 ratio and thus defective non-canonical NF-κB signaling (74). Therefore, it seems plausible that TNFa-TNFR2 interactions between Teffs and local resident cells are key to pathogenicity in colitis. The balance between distinct cellular sources of TNFa in each disease setting may influence the outcome of the immune response.

Multiple studies have shown the role of TNFa in driving immune pathology. Local expression of TNFa in neonatal non-obese diabetic (NOD) mouse islets causes an influx of antigen-specific Teffs that precedes the onset of diabetes (75). Nevertheless, TNFa can have a dichotomous role in the pathogenesis of diabetes, depending on the stage of the ongoing autoimmune process. In a transgenic model of virally induced diabetes, early islet-specific TNFa expression augmented diabetes incidence, while late TNFa expression abrogated diabetes (76). It is unclear whether TNFa acts *via* induction of autoreactive cell apoptosis or promoting the expansion of Tregs at the later stage of the immune response. In that vein, seminal work indicated that Tregs accumulated preferentially in the pancreatic lymph nodes and islets suppressed islet destruction by CD8^+^ T cells in a TNFa signaling-dependent fashion (77). Another important temporal parameter relating to the diabetogenic potential of TNFa is the duration of exposure; chronic exposure of diabetogenic T cell clones to TNFa results in T cell unresponsiveness (78).

TNFa regulates multiple aspects of Teff fate, including survival, activation, and proinflammatory cytokine production (79–81). TNFa signaling lowers the threshold for TCR-dependent activation by providing TNFR2-mediated costimulation (82). Furthermore, TNFR2 signaling delivers an anti-apoptotic stimulus during the primary T cell response, expanding the resulting memory T cell pool following a second antigen encounter (83). A clinical study showed that infliximab, a TNFa blocking antibody, induces apoptosis in activated T cells isolated from the lamina propria of steroid-refractory Crohn’s disease patients, presumably due to anti-apoptotic NF-κB signaling withdrawal (84). Lastly, TNFa signaling promotes Teff proliferation even in the presence of Treg-mediated suppression (85). However, prolonged TNFa exposure restored Treg suppressive function, suggesting that TNFa promotes an effective immune response early on, yet delivers a delayed immunoregulatory feedback signal to Tregs to restore homeostasis (85).

## Effect on Tregs in the periphery

### Thymic derived T regulatory cells (tTregs)

The inflammatory microenvironment poses several challenges to Treg stability (86). In the synovial fluid of RA patients, TNFa was found to compromise Treg suppressive capacity *via* dephosphorylation of a serine residue in the DNA-binding domain of FOXP3 (87). Several studies have focused on TNFa blocking agents. Infliximab has been reported to reverse TNFa-induced suppressive capacity and FOXP3 expression loss in Tregs (88). Adalimumab, but not etanercept, was shown to increase Treg numbers in the synovial fluid of RA patients and improve Tregs’ capacity to suppress IL-17 production (89). Interestingly, adalimumab, in contrast to etanercept, stabilizes tmTNFa on the surface of monocytes; tmTNFa then promotes Treg expansion *via* TNFR2-mediated IL-2/STAT5 signaling (89). Promoting tolerogenic tmTNFa signaling, while neutralizing sTNFa deleterious actions, may underly the increased efficiency of adalimumab over etanercept and warrants confirmation in larger clinical trials.

Multiple studies have shown that TNFR2 signaling safeguards Treg stability under inflammatory conditions. Adoptively transferred TNFR2-deficient Tregs were unable to confer protection from colitis induced by co-transferred Teffs in Rag1 KO mice, underlining the critical role of TNFR2 in Treg phenotypic and functional stability in inflammatory environments (90). Indeed, TNFR2 expression defines a maximally suppressive Treg subgroup within effector Tregs (eTreg) that accounts for tumor-infiltrating eTreg-mediated immune surveillance evasion by solid tumors (91). Conditional TNFR2 ablation in Tregs led to exacerbated Th17-mediated experimental autoimmune encephalomyelitis (EAE), a mouse model of MS, due to impaired Treg homeostasis (92). TNFa signaling has been shown to be pivotal for maintenance of eTreg phenotype in the periphery (93) and endows Tregs with the ability to suppress IFNg production by Teffs in a TNFR2-dependent manner (94).

In addition to safeguarding Treg lineage under inflammatory conditions, TNFR2 signaling synergizes with IL-2 to boost Treg proliferation *via* the non-canonical NF-κB signaling pathway (56). In a broader sense, TNFa signaling is used by Tregs to scale to inflammation. In line with this concept, Teff-derived TNFa has been shown to boost Tregs, dependent upon TNFR2 expression on Tregs, in experimental models of diabetes (95) and graft-vs-host disease (GvHD) (96). CD8^+^ T cells responding to virus infection stimulate a Vb5^+^ thymic Treg subset that expressed markers of a terminally differentiated effector cell phenotype, which promotes chronic infection in a TNFa-dependent manner (97). While proinflammatory signals enhance Treg proliferation, intense cell cycling increases the risk of Treg lineage destabilization. Indeed, IL-6 and TNFa synergistically drive robust human Treg proliferation in a TNFR2-dependent manner, with genetic deletion of TNFR2 leading to reduced expression of FOXP3 (98). TNFa-TNFR2 form a feedback loop that drives epigenetic changes that stabilize Treg phenotypic identity (99). These properties have been employed to expand Tregs *ex vivo* by delivering TNFR2 agonistic signals (100). In addition, a selective TNFR2 agonist was used to promote expansion of the endogenous Treg pool, which resulted in significantly reduced GvHD severity and mortality (101).

### Induced T regulatory cells (iTregs)

In contrast to tTregs, induced Tregs (iTregs) do not require TNFa for *in vivo* function (102). While TNFR2 KO tTregs were unable to prevent adoptive T cell transfer-induced colitis in Rag1 KO mice, TNFR2 KO iTregs were fully suppressive (102). Interestingly, pre-treatment of TNFR2 KO tTregs with TGFb restored their suppressive function (102). Even though TGFb had been previously shown to promote iTreg homeostasis in the periphery, this was the first study to show TGFb’s role in restoring TNFR2-deficient tTreg function. In fact, TNFa antagonizes TGF-b-induced iTreg generation (103).

TNFa was shown to exacerbate the development of EAE by impairing differentiation and function of iTregs *via* TNFR2-mediated activation of Akt, which in turn inhibited TGFb-induced SMAD3 phosphorylation, resulting in decreased Foxp3 expression (103). Interestingly, TNFa does not activate the Akt pathway in tTregs (103), which may explain the dichotomy of the precipitation of autoimmune side effects by anti-TNFa biologics, depending on whether tTregs or iTregs have the predominant regulatory role in the pathogenesis of each disease. In contrast, adoptive transfer of TNFR1 KO iTregs showed improved clinical scores that were associated with sustained elevated TNFR2 expression on their surface (104). Still, TNFR2 KO iTregs were unable to prevent colitis, unlike WT iTregs. The differential results of these studies may be due to differences in experimental methodology, such as the use of different genetic knockout mice, which may alter T cell development, or the use of different protocols to induce Treg differentiation.

## Therapeutic opportunities

Current TNFa blockade agents are aimed at preventing ligand binding to TNF receptors without taking into consideration the different forms of the receptor or their cell-specific expression pattern. Therefore, they indiscriminately prevent both the proinflammatory and the immunoregulatory effects of TNFR2 signaling. Novel biologics under development are aimed to selectively inhibit binding of TNFa to TNFR1, whereas selective TNFR2 activation requires both specific binding to the receptor and facilitation of oligomerization of TNFR2 complexes (105). Selective anti-TNFR1 binders under development include humanized mouse monoclonal antibodies (106), nanobodies (107), and small molecule inhibitors (108). Interestingly, XPro1595, a novel class of TNFa inhibitor called signaling-incompetent TNF derivative that inactivates sTNFa through the formation of mixed TNFa heterotrimers, has demonstrated an impressive improvement in EAE models (109). On the other hand, targeted TNFR2 binding has been achieved by generating TNFa muteins by mutagenesis or using phage display (110, 111). Fusion of homotrimeric TNF moieties allows for the generation of a nonvalent molecule capable of TNFR2 clustering (112). Indeed, sTNFa muteins S95C/G148C and TNF07, which display TNFR2 agonist properties due to their stable trimeric structure created by internal covalent cross-linking, expanded Tregs while simultaneously selectively inducing activation-induced cell death (AICD) of autoreactive CD8^+^ T cells in diabetic patients *ex vivo* (113).

## Conclusion

The outcomes of TNFa signaling in CD4^+^ T cells depend on multiple parameters, including the phase of the immune response, duration of TNFa exposure, cellular source of TNFa production, and type of TNF receptor expressed on the responding CD4^+^ T cell subpopulation. The complex role of TNFa in Treg biology holds promise in aiding the development of Treg-based therapies, both in improving *ex vivo* expansion (98) and *in vivo* function (114). Incorporating TNFa signaling in synthetic immune receptors, such as chimeric antigen receptors, could help fine tune engineered Treg therapies under development (115–117). Recent advances in our understanding of the biology of TNFa-TNFR signaling provide excellent opportunities to design targeted therapies that inhibit the effector arm of T cell immunity while unleashing the immunoregulatory properties of TNFa signaling for the treatment of autoimmune diseases and transplant rejection.

## Author contributions

NS wrote the manuscript and generated the table and the figure. LF and QT reviewed and revised the manuscript. All authors contributed to the article and approved the submitted version.

## Funding

This work was funded by Human Islet Research Network (HIRN) Emerging Leader in Type 1 Diabetes grant U24DK104162-07 (LMRF), American Cancer Society (ACS) Institutional Research Grant IRG-19-137-20 (LF), South Carolina Clinical and Translational Research (SCTR) Pilot Project Discovery Grant 1TL1TR001451-01 (LF), Diabetes Research Connection (DRC) Grant IPF 22-1224 (LF), and grants from the Beatson Foundation 2021-021 (QT) and the Northern California JDRF Center of Excellence 5-COE-2019-860-S-B (QT).

## Acknowledgments

The authors would like to thank Flavio Vincenti for helpful discussions.

## Conflict of interest

NS is an inventor in a patent application covering the use of proinflammatory cytokines in ex-vivo Treg expansion. LF is an inventor in patents and patent applications covering genetically engineered effector T cells and regulatory T cells and uses thereof. QT is a co-founder and scientific advisor of Sonoma Biotherapeutics. QT is a scientific advisor of Moderna, eGenesis, Qihan Bio, Minutia and Encellin. QT is an inventor in patents and patent applications on Treg cell therapy and Treg cell engineering.

## Publisher’s note

All claims expressed in this article are solely those of the authors and do not necessarily represent those of their affiliated organizations, or those of the publisher, the editors and the reviewers. Any product that may be evaluated in this article, or claim that may be made by its manufacturer, is not guaranteed or endorsed by the publisher.
